# Estimation of second cancer risk after radiotherapy for rectal cancer: comparison of 3D conformal radiotherapy and volumetric modulated arc therapy using different high dose fractionation schemes

**DOI:** 10.1186/s13014-016-0723-6

**Published:** 2016-11-10

**Authors:** Daniel R. Zwahlen, Laura I. Bischoff, Günther Gruber, Marcin Sumila, Uwe Schneider

**Affiliations:** 1 Department of Radiation Oncology, Kantonsspital Graubuenden, Chur, Switzerland; 2 Department of Physics, University of Zurich, Zurich, Switzerland; 3 Institute of Radiotherapy, Klinik Hirslanden, Zurich, Switzerland; 4 Department of Radiation Oncology, Kantonsspital Graubuenden, Loestrasse 170, Chur, CH-7000 Switzerland

**Keywords:** Second malignancies, Rectal cancer, Radiotherapy, Intensity-modulated radiotherapy, Volumetric-modulated arc therapy

## Abstract

**Purpose:**

To investigate second cancer risk (SCR) comparing volumetric modulated arc therapy (VMAT) and 3D conformal radiotherapy (3DCRT) with different high dose fractionation schemes.

**Methods:**

VMAT and 3DCRT virtual treatment plans for 25 patients previously treated with radiotherapy for rectal cancer were evaluated retrospectively. Doses prescribed were 25 × 1.8 Gy and 5 × 5 Gy, respectively. SCR was estimated using a carcinogenesis model and epidemiological data for carcinoma and sarcoma induction. SCR was determined by lifetime attributable risk (LAR).

**Results:**

Mean excess LAR was highest for organs adjacent to the PTV. Total LAR for VMAT and 3DCRT was 2.3–3.0 and 2.0–2.7 %, respectively. For 5 × 5 Gy, LAR was 1.4–1.9 % for VMAT and 1.2–1.6 % for 3DCRT. Organ-specific excess LAR was significantly higher for VMAT, and highest for bladder and colon. Size and shape of the PTV influenced SCR and was highest for age ≤ 40 years. For a patient with an additional lifetime risk of 60 years, LAR was 10 % for 25 × 1.8 Gy and 6 % for 5 × 5 Gy.

**Conclusions:**

No statistically significant difference was detected in SCR using VMAT or 3DCRT. For bladder and colon, organ-specific excess LAR was statistically lower using 3DCRT, however the difference was small. Compared to epidemiological data, SCR was smaller when using a hypofractionated schedule. SCR was 2 % higher at normal life expectancy.

**Trial registration:**

ClinicalTrials.gov Identifier NCT02572362. Registered 4 October 2015. Retrospectively registered.

## Introduction

Second cancer risk (SCR) is of concern in long-term radiotherapy (RT) survivors [1]. In their analysis using actuarial life-table procedures from the Uppsala Trial [2] and the Swedish Rectal Cancer Trial [3] Birgission et al. found an increased SCR in patients with rectal cancer treated with 3D conformal radiotherapy (3DCRT) in organs adjacent to the irradiated volume (RR 2.04; 95 % CI, 0.97–3.27) [4]. Wiltink et al. could not confirm an elevated SCR in a more recent series contributing to an increasingly controversial discussion [5]. Kendal and Nicholas showed in their population-based analysis from the Surveillance, Epidemiology, and End Results registry (SEER) that second cancers after RT appeared infrequently compared with background incidence of spontaneous cancers. As the median age of patients with rectal cancer is 68 years, they concluded that SCR might not be as significant compared to the therapeutic benefit and should not factor into treatment decisions for this older population [6]. However, reduction of SCR is relevant as local control increased with standard treatment including preoperative chemoradiotherapy followed by total mesorectal excision (TME) for stage II and III rectal cancer [7]. Studies have also investigated the use of a shorter course of preoperative RT (25 Gy over 5 days) without chemotherapy. It appears that short course RT results in isoeffective local control and OS compared to a long course chemoradiotherapy schedule (45 Gy over 25 days) [8]. Long term follow up of the Dutch TME trial comparing short course RT with no RT demonstrated that second malignancies were more frequent in the RT group (14 % vs. 9 %) [9]. Chemotherapy is another co-factor contributing to an increase in SCR in the population of cancer survivors. Long known to be leukemogenic, chemotherapy appears also to contribute to risk for a range of other second malignancies [10].

Recording epidemiological data on second cancers after RT for rectal cancer necessitates observation of a large group of patients over several decades. SCR is reasonably well quantified from atomic bomb survivors at moderately low doses of radiation up to 2 Gy. However, there is much more uncertainty at higher doses used in therapeutic RT [11]. We performed a model-based analysis calculating the organ-specific excess lifetime attributable risk (LAR) [12–14] to estimate SCR for patients after radiotherapy for rectal cancer comparing 3DCRT with VMAT technique.

## Methods and materials

Planning CT data of 25 patients with stage I – III rectal cancer treated with pre- or postoperative RT in 2011 to 2013 were reused for comparative treatment planning (Table 1). Patients gave informed consent to this ethically approved retrospective study (ClinicalTrials.gov Identifier: NCT02572362).

**Table 1:** Study population characteristics (*N* = 25)

Age	Years
-Mean, SD	64.8 ± 8.2
-Median (min/max)	64 (46–83)
Gender	Number of patients (%)
-M	13 (52)
-F	12 (48)
Stage (AJCC)	
I	2 (8)
IIA-B	3 (12)
IIIA-C	20 (80)
RT	
-Preoperative	20 (80)
-Postoperative	5 (20)
PTV volume (cm^3^)	
<700	6 (24)
700–1000	13 (52)
>1000	6 (24)
Monitor units (MU)	Mean, SD
−3DCRT	269.5 ± 15.1
-VMAT	512.7 ± 40.1

*SD* standard deviation, *AJCC* American Joint Committee on Cancer, *RT* Radiotherapy, *VMAT* volumetric modulated arc therapy, *3DCRT* 3 dimensional conformal radiotherapy

### Volume segmentation

Clinical target volumes (CTV) were delineated corresponding to the radiation therapy oncology group (RTOG) consensus panel contouring atlas [15]. Organs of interest with respect to cancer induction were contoured on each CT data set according to the International Commission on Radiological Protection (ICRP) 2007 [16].

### Treatment planning

For the planning target volume (PTV), an isotropic 5 mm margin was added to the CTV. 3DCRT consisted of a three-field technique - the 6 MV photon beam energy for the posteroanterior fields and 15 MV for the lateral fields were used. For five patients, 15 MV beams were applied in all three fields for anatomical reasons. Dynamic wedges were used to optimize dose distribution. For VMAT plans, photon beam energy was 6 MV using a dynamic multileaf collimator technique and one rotation per fraction. Dose rates were up 600 MU/min at maximum for both techniques. Doses prescribed were 45.0 Gy in 1.8 Gy and 25 Gy in 5 Gy per fraction, respectively. Treatment plans were normalised such that the 95 %-isodose was encompassing 98 % of the PTV volume. Eclipse External Beam Planning system version 10.0 (Varian Oncology Systems, Palo Alto, CA) and the AAA-algorithm (version 10.0.28) was used for treatment planning. Differential dose volume histograms (DVH) were generated for each plan.

### Estimation of second cancer risk

The carcinogenesis model used to estimate SCR emphasizes cell kinetics of radiation-induced cancer by mutational processes and is reported in detail elsewhere [14, 17, 18]. Briefly, the model describes carcinoma and sarcoma induction after fractionated RT as an analytical function and integrates cell sterilization processes described by the linear-quadratic model and repopulation effects. The linear-quadratic model of cell kill is applied to normal tissues that are irradiated during RT. Tumor induction is modelled such that each transformation process results in a tumor cell. Cancer induction in this model is a function of treatment dose, dose per fraction, defined cell kill parameters, tumor induction variable and repopulation parameter [17]. The obtained dose-response relationship for carcinoma and sarcoma induction can be used in models for predicting radiation-induced cancer after RT such as the organ-equivalent dose (OED) model [14]. The model parameters used in this work were obtained by fits to several epidemiological, cancer specific carcinogenesis data for carcinoma and sarcoma induction. Radiation induced cancer estimates were determined with the obtained model parameters from the publication by Schneider et al. [13]. Soft tissue sarcoma induction was estimated on the basis of the DVH for all normal tissues without the segmented organs and bone. For bone sarcoma induction, the DVH of the complete bone structure was used. The sarcoma-induction model is based on qualitative observations, like the vanishing sarcoma risk at low dose (A-bomb survivors) and the larger risk at high dose levels (RT patients) [13, 19]. The quality of epidemiological data is not strong enough to determine all model parameters. Therefore, sarcoma risk is given for three different regeneration/repopulation rates: *R* = 0.1, 0.5 and 1 which represent no, intermediate and full repopulation.

From the DVHs of structures of interest, cancer risk was estimated in terms of organ equivalent dose (OED) [14]. OED is proportional to cancer risk and was converted to excess absolute risk for a western population for each organ as well as for all organs together [17]. Lifetime cancer risk for a patient was determined by LAR according to Kellerer et al. [12] by an integration of excess absolute risk from the age at exposure to the lifetime expectancy. LAR is a lifetime risk and not applicable to epidemiological studies which include subjects with limited follow-up time. Therefore, cumulative risk is determined for these patients by taking into account the follow-up time of Birgisson et al. [4] instead of the life expectancy.

The epidemiological data are usually given in absolute risk, the modelled risk however in excess absolute risk. Therefore, the model of the base line risks was fitted to the epidemiological obtained SCR data of Birgisson et al. [4]. The sum of modelled base line risk and excess absolute risk can then be compared to the epidemiological risk found by Birgisson et al. [4].

### Statistical analysis

For every treatment plan differential DVHs were exported from the treatment planning software Eclipse External Beam Planning system version 10.0 (Varian Oncology Systems, Palo Alto, CA). Monitor units (MU), total dose and dose prescribed per fraction; PTV size and volumes of the organs of interest, age at exposure and gender were recorded. Statistical analysis was performed with R, version 3.0.2 (2013-09-25), (R Foundation for Statistical Computing, Vienna, Austria). Median, mean values and standard deviation of the mean (SD) were collected. Student’s *T*-test for paired samples and nonparametric Wilcoxon signed-rank test were used. A *p* value < 0.05 was considered statistically significant. Confidence intervals (CI) included 95 % of the measured data.

## Results

Mean percentage excess LAR for segmented organs, bones or soft tissues as well as sex specific LAR are shown in Table 2. Analysis was performed using actual age of patients at the time of RT. LAR was integrated up to an attained age of 90 years.

**Table 2:** Excess lifetime attributable risk (LAR) after RT

Organ^a^	Mean LAR VMAT (%)		Mean LAR 3DCRT (%)	
	25 × 1.8 Gy	5 × 5 Gy	25 × 1.8 Gy	5 × 5 Gy
Anus	0.3330	0.1973	0.3098	0.1835
Bladder	0.2151	0.2260	0.1067	0.1144
Bones R = 1	0.1734	0.0881	0.1756	0.0864
Bones R = 0.5	0.0591	0.0273	0.0573	0.0257
Bones R = 0.1	0.0031	0.0013	0.0029	0.0013
Colon	1.0225	0.5677	0.8554	0.4754
Sigmoid	0.4732	0.2882	0.4981	0.3020
Skin	0.0519	0.0288	0.0461	0.0256
Small bowel	0.1328	0.1088	0.1240	0.0977
Soft tissue R = 1	0.1007	0.0474	0.0768	0.0357
Soft tissue R = 0.5	0.0344	0.0153	0.0254	0.0115
Soft tissue R = 0.1	0.0024	0.0011	0.0019	0.0008
Prostate	0.0354	0.0242	0.0346	0.0236
Ovaries	0.2756	0.1944	0.2838	0.2053
Uterus	0.1997	0.1383	0.1826	0.1220
All organs male R = 1	2.5380	1.5764	2.2270	1.3443
All organs male R = 0.5	2.3574	1.4835	2.0573	1.2593
All organs male R = 0.1	2.2694	1.4433	1.9795	1.2242
All organs female R = 1	2.9778	1.8849	2.6589	1.6480
All organs female R = 0.5	2.7972	1.7920	2.4892	1.5630
All organs female R = 0.1	2.7093	1.7518	2.4113	1.5279

*VMAT* volumetric modulated arc therapy, *3DCRT* 3 dimensional conformal radiotherapy, *R* regeneration rate for soft tissue and bones, with possible sarcoma induction

^a^female/male data mixed if not otherwise indicated

Mean excess LAR was highest for organs adjacent or close to the PTV (Table 2). For all organs using 25 × 1.8 Gy, the LAR for VMAT and 3DCRT was 2.3 – 3.0 and 2.0 – 2.7 %, respectively (Table 2). For 5 × 5 Gy, LAR was 1.4 – 1.9 % for VMAT and 1.2 – 1.6 % for 3DCRT and half as high as for the long course RT scheme (Table 2). Comparing VMAT with 3DCRT, median percentage excess LAR difference for bladder, colon, anus, small bowel, soft tissue and skin was significantly higher for VMAT irrespective of fractionation (Table 3). Percentage excess LAR difference was highest for bladder and colon. Excess LAR for sarcomas was small compared to carcinoma independent of which repopulation rate was used.

**Table 3:** Excess lifetime attributable risk (LAR) VMAT vs 3DCRT

Organ^a^	25 × 1.8 Gy (VMAT > LAR)			5 × 5 Gy (VMAT > LAR)		
	Median (%)	95 % CI	*p*-value	Median (%)	95 % CI	*p*-value
Anus	0.0145	0.0016–0.0406	0.016	0.0088	0.0013–0.0241	0.013
Bladder	0.0934	0.0486–0.1491	<0.001	0.0988	0.0568–0.1572	<0.001
Bones R = 1	0.0066	−0.0223–0.0171	0.458	0.0057	−0.0089–0.0114	0.312
Bones R = 0.5	0.0040	−0.0057–0.0080	0.287	0.0026	−0.0019–0.0046	0.220
Bones R = 0.1	0.0003	−0.0002–0.0006	0.202	0.0001	−0.0001–0.0003	0.182
Colon	0.0768	0.0097–0.2674	0.015	0.0417	0.0051–0.1474	0.012
Sigmoid	0.0054	−0.0077–0.0152	0.367	0.0040	−0.0036–0.0106	0.241
Skin	0.0050	0.0027–0.0073	<0.001	0.0028	0.0014–0.0040	<0.001
Small bowel	0.0064	0.0137–0.0259	<0.001	0.0094	0.0052–0.0147	<0.001
Soft tissue R = 1	0.0195	0.0137–0.0259	<0.001	0.0094	0.0021–0.0042	<0.001
Soft tissue R = 0.5	0.0072	0.0050–0.0097	<0.001	0.0031	0.0050–0.0097	<0.001
Soft tissue R = 0.1	0.0005	0.0003–0.0006	<0.001	0.0002	0.0001–0.0003	<0.001
Prostate	0.0002	−0.0026–0.0043	0.787	0.0001	−0.0024–0.0034	0.893
Uterus	0.0089	0.0055–0.0555	0.063	0.0077	0.0042–0.0546	0.063

*VMAT* volumetric modulated arc therapy, *3DCRT* 3 dimensional conformal radiotherapy, *R* regeneration rate for soft tissue and bones, with possible sarcoma induction, *CI* confidence interval

^a^female/male data mixed if not otherwise indicated

**Fig. 1:**
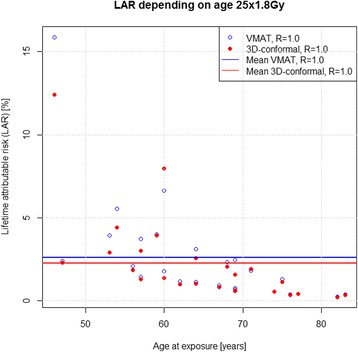
LAR for each patient with RT 25 × 1.8 Gy, R = 1

**Fig. 2:**
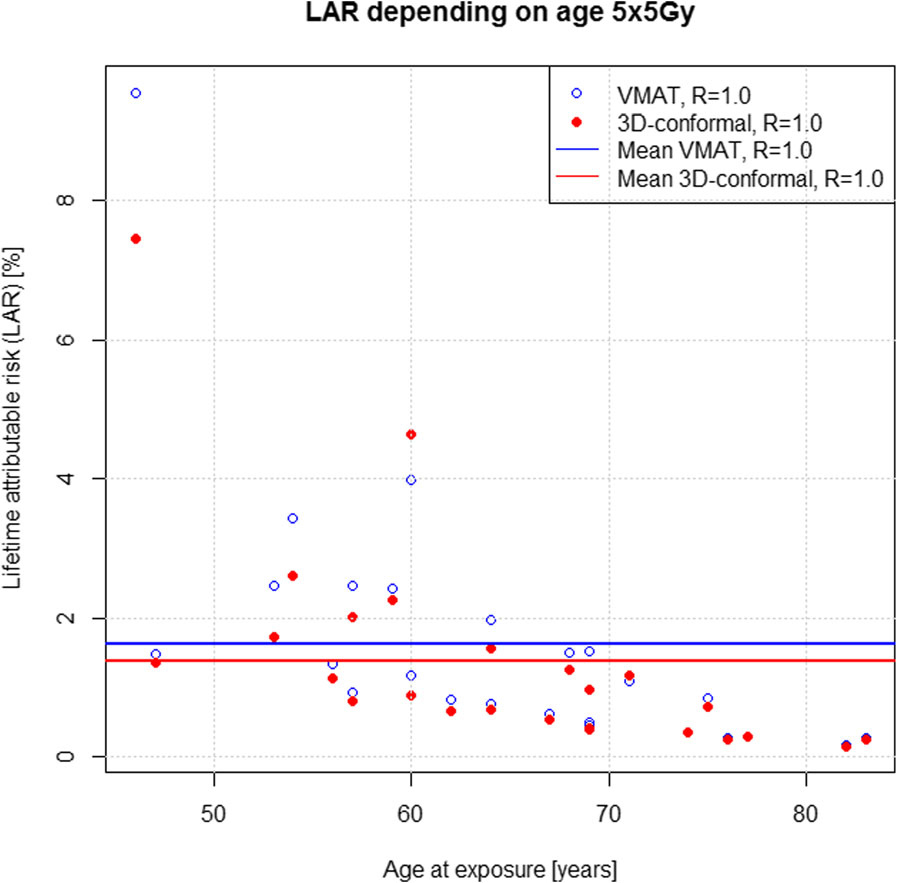
LAR for each patient with 5 × 5 Gy, R = 1

Large patient specific differences in excess LAR could be determined for 25 × 1.8 Gy, ranging from 15.9 % for patients younger than 50 years and 0.2 % for patients older than 80 years. Accordingly, when using the 5 × 5 Gy regimen excess LAR was 9.6 % for patients under 50 years and 0.1 % for those over 80 years (Figs. 1 and 2) and regeneration/repopulation rates (*R* = 0.1, 0.5, 1) did not influence excess LAR. Percentage excess LAR was higher for VMAT for patients younger than 60 years. However, mean excess LAR was only 2 % for the long course RT and 1.5 % for the short course regimen using either technique. Therefore, SCR could not only be explained by age at exposure, but also by other factors including size and shape of the target volume.

**Fig. 3:**
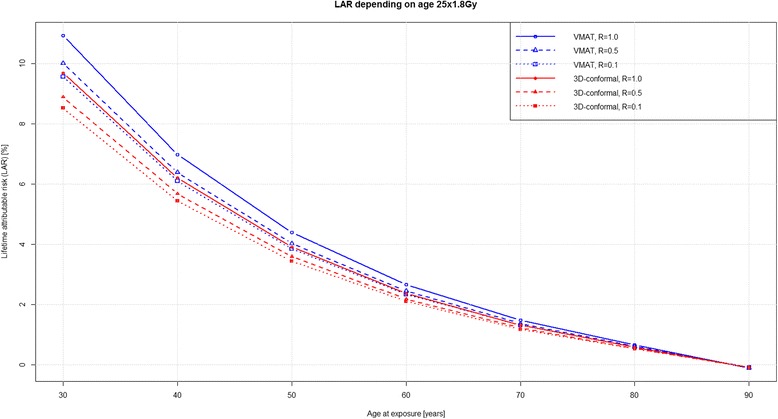
LAR with a variable age at exposure and attained age of 90 years for RT 25 × 1.8 Gy

**Fig. 4:**
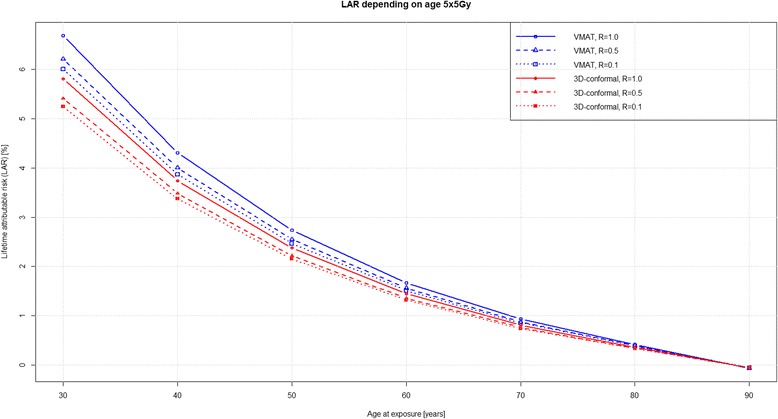
LAR with a variable age at exposure and attained age of 90 years for 5 × 5 Gy

Figures 3 and 4 show excess LAR for patients at different ages of exposure and attained age of 90 years. SCR was highest for patients younger than 40 years of age. According to our model, a 30-year-old irradiated patient with an additional lifetime of 60 years, has an excess LAR of 10 % for 25 × 1.8 Gy and 6 % for 5 × 5 Gy. LAR difference decreased with age between the long and short course schedules. For patients 65 – 70 years of age and an age most commonly diagnosed and treated for rectal cancer, calculated second cancer risk difference was 0.3 % and statistically non-significant, irrespective of RT regimen and technique used.

**Fig. 5:**
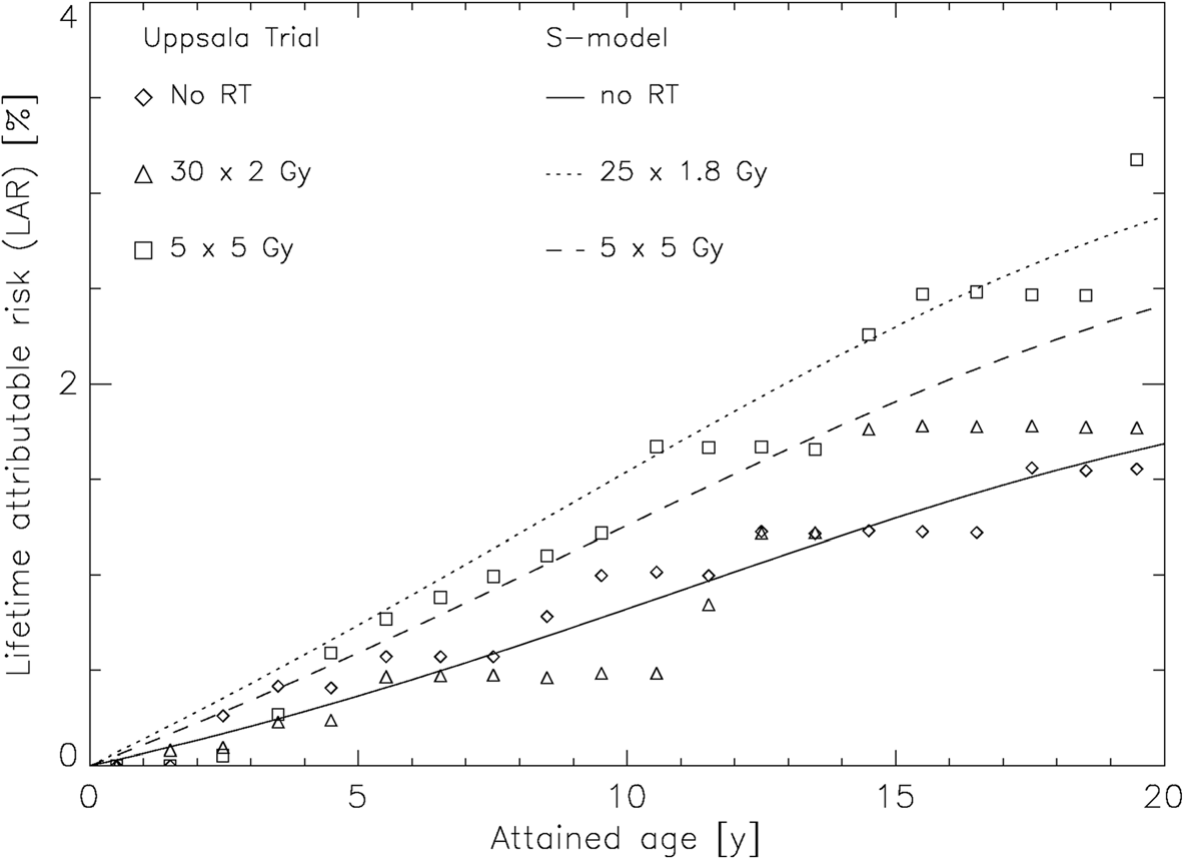
LAR for the patients treated with 30 × 2 Gy and 5 × 5 Gy (Uppsala Trial) [4, 20] as well as 25 × 1.8 Gy and 5 × 5y (S-model) [17] over 20 years (age 69 – 89) and no RT

To estimate accuracy of the S-model [17] used in this work, we compared our results with epidemiological data reported by Birgisson et al. [4]. LAR calculations were performed for an age at RT exposure of 69 years (corresponding to median age at diagnosis of rectal cancer in the study of Birgisson et al. [4]), and integrated up to an attained age of 89 years (Fig. 5). SCR for rectal cancer patients was modelled using the background rate in the patient cohort analysed by Birgisson et al. [4]. Modelled baseline risk was added to the calculated excess LAR of patients treated with 3DCRT and 28 ×1.8 Gy and 5 × 5 Gy (Figs. 1 and 2), respectively. Baseline LAR was 1.7 % after 20 years of follow-up. The modelled excess LAR for a 2 Gy and 5 Gy fractionation schedule was 7 and 43 % of the baseline-LAR, respectively. Gradients of the attained age increasing risks were similar for both the S-model [17] and the epidemiological data. Absolute LAR at 20 years of follow-up was compared. For the Uppsala Trial [20] (30 × 2 Gy) absolute LAR was 1.8 % compared to 2.9 % for the S-model [17] (25 × 1.8 Gy), and 3.2 % versus 2.4 % for the Uppsala Trial [20] and S-model [17] using 5 × 5 Gy.

## Discussion

Similar to static multi-field intensity-modulated RT (IMRT) use of VMAT results in superior target coverage and OAR sparing than 3DCRT, reducing treatment-induced toxicity, and has the potential to increase tumor control in rectal cancer [21, 22]. However, larger volumes of normal tissues are exposed to low dose ionizing radiation using VMAT due to its continuous delivery while the arc is rotating around the patient [23, 24]. In this study, it was postulated that SCR differs using VMAT while delivering a larger low dose bath to the pelvis and more MU than with a three- or four-field 3DCRT box technique. There are several reasons that could explain why we did not find a difference in SCR comparing the two techniques. First, beam on time is often longer with VMAT, thus increasing dynamic multileaf collimator interleaf leakage as well as collimator scatter, known to increase SCR [23, 25–27]; conversely, use of hard or dynamic wedges with 3DCRT increases beam on time and MU, neutralizing the advantage of 3DCRT with respect to shorter beam on time. Secondly, dose coverage of a horseshoe-shaped PTV is more conformal with VMAT resulting in a smaller high dose volume. As a consequence, adjacent OAR are less exposed to higher doses [28]. As is known from other studies, SCR is highest in tissues or organs that are closely located to or at the margins of the PTV [29, 30]; hence, a smaller PTV potentially results in a decrease of SCR. This fact may even compensate for the larger low dose bath to the pelvis with VMAT otherwise responsible for higher SCR [23, 31]. Thirdly, in our study MU count was higher with VMAT compared to 3DCRT (Table 1). As the treated volume and thus scatter dose was smaller, higher number of MU with VMAT did not translate automatically into higher SCR. The fact that with a highly conformal RT technique such as VMAT SCR can be reduced is noted by the work of Rehman et al. reporting on lowest SCR with VMAT in spine radiotherapy compared to 3DCRT and IMRT [28].

Most second cancers occur in organs adjacent to or near the target volume [25, 29, 30]. Our study could confirm this observation as higher LAR was found for organs close to the PTV including colon, sigmoid, anus and bladder irrespective of RT technique or fractionation scheme (Tables 2 and 3). Comparing the RT techniques used in this study, organ-specific LAR was significantly lower using 3DCRT than VMAT. This is in contrast to the studies by Rehman et al. and Mok et al., both reporting on lower doses to OAR close to the PTV using either 6 MV VMAT or 6 MV IMRT [21, 28]. This contradiction could be explained by the fact that despite using 6 MV VMAT, irradiated volumes for rectal cancer and for spine metastases are very different in size [28] neutralizing the advantage of a highly conformal treatment technique for the PTV with larger field sizes and adding a larger low dose bath to organs around the target volume with VMAT compared to 3DCRT [23]. It should also be noted that when using 3DCRT to treat rectal cancer, beam energy used is usually larger than 10 MV, and as a consequence production of secondary neutrons occurs, contributing to increased SCR [25, 32].

Second malignancies after fractionated RT are of increasing concern and influenced by cellular repopulation during and shortly after treatment [33]. The influence of fraction size on cellular repopulation favoring second cancer induction is only partly understood. Sachs et al. postulate in their stochastic initiation/inactivation/proliferation (IIP) model that fractionated higher dose RT leads to a growth advantage for pre-malignant cells and therefore explains the increase of the year-specific excess relative SCR incidence for a specific organ. Taking into account the weekend treatment gaps this effect is adversely related to SCR, as repopulation during weekends tends to increase the number of pre-malignant cells [33]. These conclusions would favor hypofractionated RT regimens in reducing SCR, as seen in our study. Manem et al. showed in their work modeling tumor control probability and tumor recurrence time on the basis of the linear quadratic model and SCR on the basis of the IIP model [11] that with hypofractionation, SCR was reduced by 22 % compared to a conventional (2Gy) fractionation schedule [34]. Schneider et al. also reported that SCR decreases linearly by around 10–15 % per 1 Gy for both carcinoma and sarcoma induction with increasing fractionation dose, and using conventional rather than stereotactic RT, where this effect has not been detected [35]. However, these findings, including our own data based on the S-model [17], are in contradiction to the epidemiological data by Birgisson et al. [4] showing a trend towards a higher SCR using hypofractionation (Fig. 5). One explanation could be that in the models used so far, results were based on other histologies than seen in rectal cancer and the effect of different fractionation is unknown. Edmondson et al. demonstrated in their mouse model that tumor induction using either a conventional or single fractionation regimen was dependent on tumor histology and hence genetic susceptibility, and in particular non-sarcomatous second cancer were seen more frequently after exposure to RT in fractions of 2 Gy per day [36]. Our group was able to show that sterilization of a large number of cells at higher doses could lead to inflammation or proliferative stress and initiate carcinogenesis. This might explain why the models discussed so far incorrectly assume a decrease of SCR through hypofractionation. In particular, at doses > 20 Gy cancer induction could be systematically underestimated in the current mutational models, as tissue injury due to high doses of radiation may be due to enhanced cell proliferation escaping senescence and apoptosis [37]. However, the impact of SCR on accelerated carcinogenesis after RT at different dose levels remains an area for future research.

Compared to the detected increase in SCR of 2 % for either technique, RT-induced cancer risk remains low compared to spontaneous cancer induction for the older patient population included in this study (Fig. 3 and 4). Therefore, the question remains how relevant is the detected increase for treatment decision making and does it potentially harm cancer survivors. Tubiana stated that the incidence of second primary malignancies had long been underestimated due to the short life expectancy of most cancer patients. However, with improvement of long-term survival due to better treatment results, second primary malignancies became relevant as the delay between RT and second cancer may be as long as 10 years or more. Other factors influencing SCR include type of tissue or organ, age of patient at treatment, hereditary factors, but also RT volume and dose [1]. These findings have been confirmed by Hodgson et al. in young female Hodgkin lymphoma survivors, where treatment field size and dose substantially influenced SCR for consecutive breast and lung cancer 11-fold and 3.6-fold, respectively [38]. This is in contrast to the SCR found in this work, indicating that in an older patient population second primary malignancies for rectal cancer after RT occurred relatively infrequently compared to spontaneously occurring malignancies [5, 6].

A limitation of our study was that the results were based on a mutational model and uncertainties were involved in modeling the underlying biology of radiation-induced cancer. As very little is known about the shape of the dose-response relationship for radiation-induced cancer in the RT dose range currently applied, our approach could be used to investigate at least quantitatively fractionation dependence of second cancer induction. It should also be noted that in addition to the limitations mentioned, the S-model [17] is a carcinogenesis model without acceleration and therefore time-related effects independent of RT dose, such as the delayed start of repopulation, were neglected [30]. Finally, the small number of patients used for the present analysis constitutes another limitation of the study.

## Conclusion

In conclusion, this study showed no statistically significant difference in SCR using either VMAT or 3DCRT. Organ-specific LAR to develop a treatment-related second cancer was higher with VMAT compared to 3DCRT and highest for organs at risk neighbouring the PTV. Compared to spontaneous cancer induction, radiation-induced cancer risk was low. For both techniques, SCR was approximately 2 % for the typical age (69 years) at exposure. However, SCR could increase by 10 % for a patient at 30 years of age, and SCR increased exponentially with decreasing age at exposure to RT.

## References

[1] Tubiana M (2009). Can we reduce the incidence of second primary malignancies occurring after radiotherapy? A critical review. Radiother Oncol.

[2] Frykholm GJ, Glimelius B, Pahlman L (1993). Preoperative or postoperative irradiation in adenocarcinoma of the rectum: final treatment results of a randomized trial and an evaluation of late secondary effects. Dis Colon Rectum.

[3] Trial SRC (1997). Improved survival with preoperative radiotherapy in resectable rectal cancer. Swedish Rectal Cancer Trial. N Engl J Med.

[4] Birgisson H (2005). Occurrence of second cancers in patients treated with radiotherapy for rectal cancer. J Clin Oncol.

[5] Wiltink LM (2015). No increased risk of second cancer after radiotherapy in patients treated for rectal or endometrial cancer in the randomized TME, PORTEC-1, and PORTEC-2 trials. J Clin Oncol.

[6] Kendal WS, Nicholas G (2007). A population-based analysis of second primary cancers after irradiation for rectal cancer. Am J Clin Oncol.

[7] Sauer R (2012). Preoperative versus postoperative chemoradiotherapy for locally advanced rectal cancer: results of the German CAO/ARO/AIO-94 randomized phase III trial after a median follow-up of 11 years. J Clin Oncol.

[8] Glimelius B (2013). Rectal cancer: ESMO Clinical Practice Guidelines for diagnosis, treatment and follow-up. Ann Oncol.

[9] van Gijn W (2011). Preoperative radiotherapy combined with total mesorectal excision for resectable rectal cancer: 12-year follow-up of the multicentre, randomised controlled TME trial. Lancet Oncol.

[10] Morton LM et al. The rising incidence of second cancers: patterns of occurrence and identification of risk factors for children and adults. Am Soc Clin Oncol Educ Book. 2014:e57-67. doi:10.14694/EdBook_AM.2014.34.e57. 10.14694/EdBook_AM.2014.34.e57 24857148

[11] Sachs RK, Brenner DJ (2005). Solid tumor risks after high doses of ionizing radiation. Proc Natl Acad Sci U S A.

[12] Kellerer AM, Nekolla EA, Walsh L (2001). On the conversion of solid cancer excess relative risk into lifetime attributable risk. Radiat Environ Biophys.

[13] Schneider U, Sumila M, Robotka J (2011). Site-specific dose-response relationships for cancer induction from the combined Japanese A-bomb and Hodgkin cohorts for doses relevant to radiotherapy. Theor Biol Med Model.

[14] Schneider U (2005). Estimation of radiation-induced cancer from three-dimensional dose distributions: concept of organ equivalent dose. Int J Radiat Oncol Biol Phys.

[15] Myerson RJ (2009). Elective clinical target volumes for conformal therapy in anorectal cancer: a radiation therapy oncology group consensus panel contouring atlas. Int J Radiat Oncol Biol Phys.

[16] Protection, I.C.o.R (2007). The 2007 recommendations of the international commission on radiological protection. ICRP publication 103. Ann ICRP.

[17] Schneider U (2009). Mechanistic model of radiation-induced cancer after fractionated radiotherapy using the linear-quadratic formula. Med Phys.

[18] Schneider U, Kaser-Hotz B (2005). Radiation risk estimates after radiotherapy: application of the organ equivalent dose concept to plateau dose-response relationships. Radiat Environ Biophys.

[19] Preston DL (2007). Solid cancer incidence in atomic bomb survivors: 1958–1998. Radiat Res.

[20] Pahlman L, Glimelius B, Graffman S (1985). Pre- versus postoperative radiotherapy in rectal carcinoma: an interim report from a randomized multicentre trial. Br J Surg.

[21] Mok H (2011). Intensity modulated radiation therapy (IMRT): differences in target volumes and improvement in clinically relevant doses to small bowel in rectal carcinoma. Radiat Oncol.

[22] Richetti A (2010). Neo-adjuvant chemo-radiation of rectal cancer with volumetric modulated arc therapy: summary of technical and dosimetric features and early clinical experience. Radiat Oncol.

[23] Kjaer-Kristoffersen F (2009). RapidArc volumetric modulated therapy planning for prostate cancer patients. Acta Oncol.

[24] Rechner LA (2012). Risk of radiogenic second cancers following volumetric modulated arc therapy and proton arc therapy for prostate cancer. Phys Med Biol.

[25] Hall EJ, Wuu CS (2003). Radiation-induced second cancers: the impact of 3D-CRT and IMRT. Int J Radiat Oncol Biol Phys.

[26] Lillicrap SC, Morgan HM, Shakeshaft JT (2000). X-ray leakage during radiotherapy. Br J Radiol.

[27] Pasler M, Wirtz H, Lutterbach J (2011). Impact of gantry rotation time on plan quality and dosimetric verification--volumetric modulated arc therapy (VMAT) vs. intensity modulated radiotherapy (IMRT). Strahlenther Onkol.

[28] Rehman J (2015). Evaluations of secondary cancer risk in spine radiotherapy using 3DCRT, IMRT, and VMAT: A phantom study. Med Dosim.

[29] Boice JD (1985). Second cancers following radiation treatment for cervical cancer. An international collaboration among cancer registries. J Natl Cancer Inst.

[30] Dorr W, Herrmann T (2002). Second primary tumors after radiotherapy for malignancies. Treatment-related parameters. Strahlenther Onkol.

[31] Hall EJ (2006). Intensity-modulated radiation therapy, protons, and the risk of second cancers. Int J Radiat Oncol Biol Phys.

[32] Howell RM (2006). Calculation of effective dose from measurements of secondary neutron spectra and scattered photon dose from dynamic MLC IMRT for 6 MV, 15 MV, and 18 MV beam energies. Med Phys.

[33] Sachs RK (2007). Second cancers after fractionated radiotherapy: stochastic population dynamics effects. J Theor Biol.

[34] Manem VS (2014). Efficacy of dose escalation on TCP, recurrence and second cancer risks: a mathematical study. Br J Radiol.

[35] Schneider U, Besserer J, Mack A (2010). Hypofractionated radiotherapy has the potential for second cancer reduction. Theor Biol Med Model.

[36] Edmondson EF (2015). Tumor Induction in Mice After Localized Single- or Fractionated-Dose Irradiation: Differences in Tumor Histotype and Genetic Susceptibility Based on Dose Scheduling. Int J Radiat Oncol Biol Phys.

[37] Schneider U, Schafer B (2012). Model of accelerated carcinogenesis based on proliferative stress and inflammation for doses relevant to radiotherapy. Radiat Environ Biophys.

[38] Hodgson DC (2007). Individualized estimates of second cancer risks after contemporary radiation therapy for Hodgkin lymphoma. Cancer.

